# Prognostic factors of papillary and follicular carcinomas based on pre-, intra-, and post-operative findings

**DOI:** 10.1530/ETJ-24-0196

**Published:** 2024-10-04

**Authors:** Yasuhiro Ito, Akira Miyauchi

**Affiliations:** 1 Department of Surgery, Kuma Hospital, Shimoyamate-dori, Chuo-ku, Kobe, Hyogo, Japan

**Keywords:** differentiated thyroid carcinoma, follicular carcinoma, papillary carcinoma, prognostic factors

## Abstract

**Graphical abstract:**

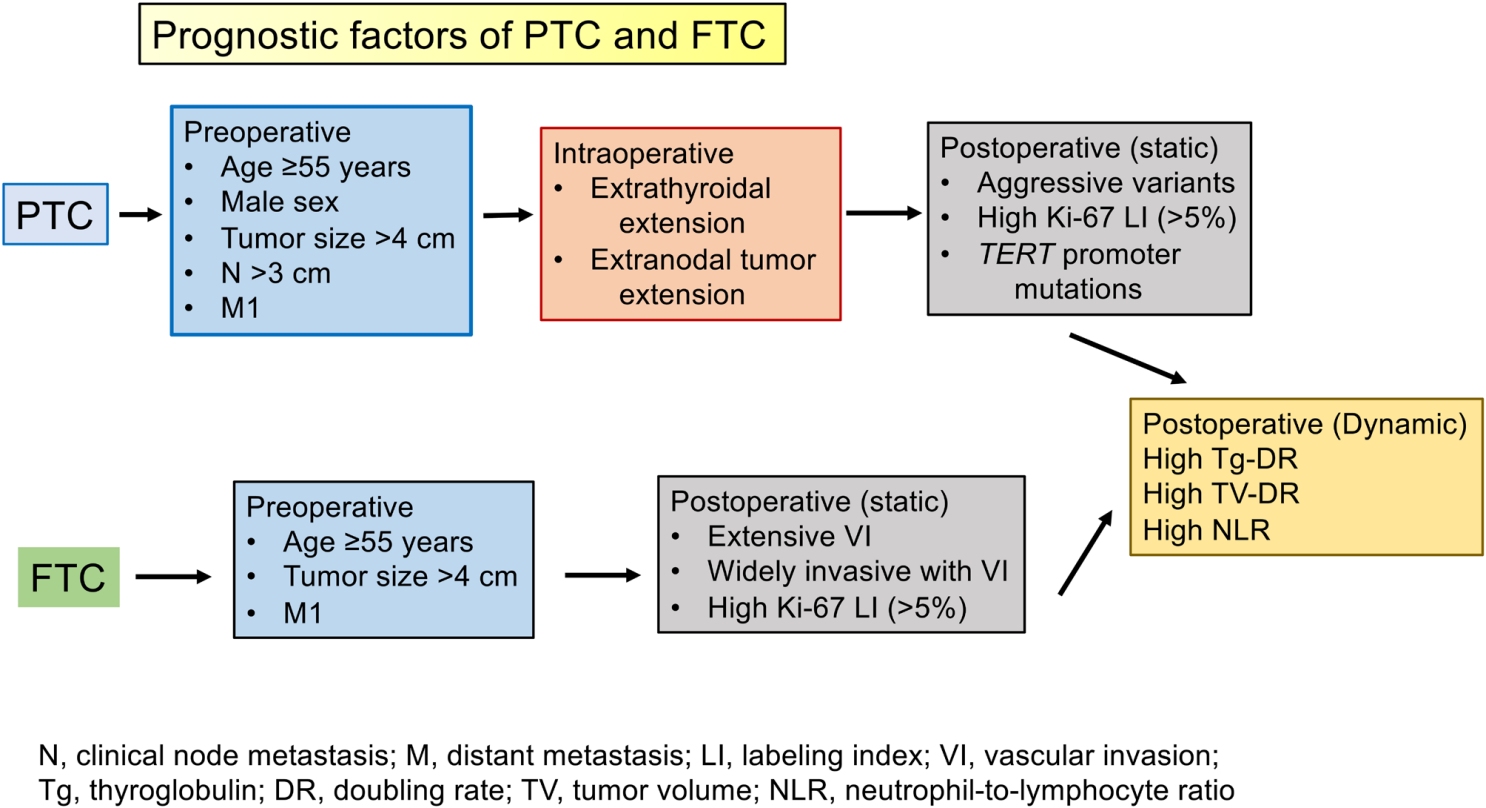

**Abstract:**

Papillary and follicular thyroid carcinomas (PTC and FTC) are prominent malignancies that originate from thyroid follicular cells. PTC is usually diagnosed via preoperative cytology, and large tumor size, clinical node metastasis, and distant metastasis constitute preoperative prognostic factors. Gross extrathyroidal and extranodal tumor extensions have a significant prognostic impact, are evaluated intraoperatively, and are useful for determining the extent of surgery. Aggressive variants, such as tall cell and hobnail variants, a high Ki-67 labeling index (LI), and somatic gene mutations are prognostic factors in postoperative pathological and molecular examinations. In contrast, FTC is generally diagnosed based on postoperative pathology. Large tumor size and M factors have prognostic value; however, the findings of pathological examinations are very important. FTCs are classified as minimally invasive, encapsulated angioinvasive, and widely invasive FTCs. Widely invasive FTC with vascular invasion (VI) and encapsulated angioinvasive FTCs with extensive VI have a poor prognosis, whereas widely invasive FTC without VI has an excellent prognosis, which is similar to that of minimally invasive FTC. This indicates that VI is a considerably more important prognostic marker than capsular invasion. For postoperative follow-up, dynamic markers such as the thyroglobulin-doubling rate (DR), metastatic tumor volume-DR, and change in the neutrophil-to-lymphocyte ratio are important and are useful for evaluating the effectiveness of treatments, such as radioactive iodine therapy and molecular targeted therapy, for recurrent lesions. For clinicians, it is important to accurately evaluate prognostic markers of PTC and FTC in the pre-, intra-operative, and post-operative phases.

## Introduction

Papillary thyroid carcinoma (PTC) and follicular thyroid carcinoma (FTC) are major components of differentiated thyroid carcinoma (DTC) and are representative of malignancies that originate from the thyroid. Although both PTC and FTC originate from thyroid follicular cells, they have rather different characteristics. PTC frequently invades adjacent organs, such as the recurrent laryngeal nerve, esophagus, and trachea; however, this phenomenon is rare in FTC. Regarding metastases and recurrences, PTC frequently metastasizes to regional lymph nodes, whereas distant metastasis/recurrence is predominant for FTC. PTC is generally diagnosed by cytological examination, whereas FTC is normally diagnosed on postoperative pathological examination because of the difficulties associated with the cytological distinction between FTC and benign follicular adenoma.

Both carcinomas generally have a favorable prognosis if appropriately managed; however, in patients with certain clinicopathological features, the prognosis could be limited. The prognostic factors can be classified into two categories: static and dynamic. The former is further classified according to the periods when they are evaluated into three subcategories: pre-, intra-, and post-operative findings. They are useful in deciding therapeutic strategies, including the extent of surgery, adjuvant therapies such as radioactive iodine (RAI) administration, and postoperative follow-up imaging studies. Based on the 8th Edition of the American Joint Committee on Cancer (AJCC) staging (1), all data obtained within 4 months after surgery should be used for defining AJCC staging. This indicates that the ‘static period’ includes all preoperative and intraoperative findings, and postoperative findings detected up to 4 months after surgery. Furthermore, for PTC, some molecular markers, such as *BRAF* and *TERT* promoter gene mutations, have been identified. Although *BRAF* mutations are frequently detected in PTC, studies of the prognostic value of *TERT* promoter gene mutations have been published by only some institutions, especially in Japan. Dynamic factors are based on changes in blood markers and metastatic/recurrent tumors, which are useful for selecting postoperative therapies such as RAI therapy and molecular targeted therapy, to evaluate the effectiveness of these treatments, and to ascertain outcomes. In this review, we discuss both the static and dynamic prognostic factors for PTC and FTC.

Previously, there was no clear pathological distinction between PTC and FTC. However, this has changed with the reclassification of the PTC and FTC subtypes. For example, the follicular variant PTC, which was previously classified as FTC, has been reclassified as a PTC. Moreover, the invasive encapsulated follicular variant PTC has been separately classified from other PTC and FTC subtypes (1). Therefore, it is uncertain whether findings related to ‘FTC’ that were reported in previous studies (published 15–20 years ago) are relevant to our current classification system. In this review, we focused on recent studies reporting analyses of pathological specimens based on the current classification systems instituted in the previous 10–15 years.

## Static prognostic factors of PTC

### Factors based on preoperative findings

#### Age

Age is a well-known predominant prognostic factor. According to the current Tumor-Node-Metastasis (TNM) classification from the American Joint Committee on Cancer (AJCC)/Union for International Cancer Control (UICC), all patients without distant metastasis (M0) are classified as stage I if they are younger than 55 years (1). As the AJCC staging system is designed to predict death from thyroid carcinoma, several previous studies have set the cutoff age for the endpoint of prognosis at disease-specific survival (DSS; Table 1). Although, in the previous version of the AJCC staging system, the age cutoff was 45 years, in 2010, Ito *et al*. reported that an age cutoff of 55 years more clearly reflected patient prognosis, including DSS (2). After the revision of the TNM classification system from the 7th to the 8th edition, the investigators showed that, among the 5892 patients with PTC, the number of patients with stage I disease increased from 3975 (79.0%) to 5034 (85.4%) mainly because of the revised age cutoff (3). Notably, the DSS rates of stage I patients did not differ before and after revision. Nixon *et al*. and Mazurat *et al*. concluded that for the DSS of patients with DTC, an age cutoff of 55 years was more robust than that of 45 years (4, 5). Furthermore, Trimboli *et al*. showed that the application of an age cutoff of 55 years to the American Thyroid Association (ATA) stratification system could help identify patients with the highest risk of relapse for disease-free survival (DFS) (6), whereas Sugitani *et al.* reported that 50 years was the optimal age cutoff for deriving DSS using their classification system (7). Recently, Sugino *et al*. showed that the DSS of patients with PTC without extrathyroidal extension was generally good regardless of age; however, the receiver operating characteristic (ROC) curve analysis showed that an age cutoff of 48 years was optimal for identifying patients with extrathyroidal extension and predicting a poor DSS (8). Some studies have proposed the following cutoff ages for DFS and DSS: 40 and 60 years, 35 and 62.5 years, and 30 and 60 years (9, 10, 11). The investigators showed that the prognostic significance of age in PTC is actually biphasic, wherein young patients are likely to show recurrence whereas older patients are likely to experience recurrence and die of thyroid carcinoma. Miyauchi *et al*. reported high proportions of patients with biochemically persistent disease among those with PTC who underwent total thyroidectomy and were younger than 40 years or older than 60 years; however, only the latter group had a short thyroglobulin-doubling time (9). This finding is consistent with the fact that carcinoma recurrence is more likely in young and old patients, although only older age is a mortality risk factor.

**Table 1:** Cutoff age proposed in previous studies.

Studies	Optimal cutoff age (years)	Endpoint of prognosis
Ito *et al*. (2, 3)	55	DFS, DSS, OS
Nixon *et al*. (4)	55	DSS (for DTC)
Mazurat *et al*. (5)	55	DFS, DSS (for DTC)
Trimboli *et al*. (6)	55	DFS (for DTC)
Sugitani *et al*. (7)	50	DSS
Sugino *et al*. (8)	48	DSS (for PTC with ETE)
Miyauchi *et al*. (9)	40 and 60	BPD
	60	Short Tg-DT
Cho *et al*. (10)	35 and 62.5	DFS
	62.5	DSS
Ito *et al*. (11)	30 and 60	DFS
	60	DSS

BPD, biochemically persistent disease; DFS, disease-free survival; DSS, disease-specific survival; DTC, differentiated thyroid carcinoma; ETE, extrathyroidal extension; OS, overall survival; PTC, papillary thyroid carcinoma; Tg-DT, thyroglobulin doubling time.

#### Male sex

Although sex has not been incorporated into the UICC/AJCC TNM classification (1), AMES set sex-specific age cutoffs as ≤40 and ≤50 years for men and women, respectively (12). Ding *et al*. showed that PTC in male patients, compared to that in female patients, had more aggressive clinicopathological behaviors, such as large size, multiple tumors, bilateral tumors, and metastases-positive central and lateral nodes (13). Furthermore, Siraj *et al*. demonstrated that male patients had higher pT, pN, pM, and pTNM stages (14). A few single-institution studies and meta-analyses have shown that male sex is a predictor of poor prognosis (13, 14, 15, 16). In a single-institution study that enrolled 5897 patients with PTC, male sex was an independent prognostic factor for overall survival (OS) in patients aged <55 years (17).

#### Tumor size

The UICC/AJCC TNM classification has specified a 4-cm cutoff as the tumor size for upstaging of patients ≥55 years (1), and the current ATA guidelines recommend lobectomy for low-risk DTCs measuring 1–4 cm (18). Gordon *et al*. reported that, after the publication of these guidelines in 2015, the incidence of lobectomy increased from 13.7% to 22.9%, whereas that of adjuvant RAI administration decreased from 48.7% to 37.1% (19). Thus, previous clinical studies have shown that a tumor size >4 cm is a significant prognostic factor for DFS, DSS, and OS (17, 20).

Another noteworthy aspect is tumor-size-related differences in significant prognostic factors. Fukushima *et al*. reported that the prognostic significance of clinical lateral node metastasis (N1b) was higher than that of extrathyroidal extension in PTC ≤ 3.0 cm, whereas this significance was reversed in PTC > 3.0 cm (21). Ito *et al*. showed that extrathyroidal extension worsens the CSS in patients with PTC > 2 cm, but not in those with PTC ≤ 2 cm (22). Moreover, Liu *et al*. reported a higher recurrence rate of macroscopic extrathyroidal extension in PTC > 1 cm, but no significant value in PTC ≤ 1 cm (23).

#### Clinical lymph node (N1) metastasis: Importance of size of metastatic nodes

Numerous studies of the prognostic significance of N positivity have been published; however, the characteristics of N are also important. In 2004, Sugitani *et al*. evaluated the size of metastatic nodes and showed that N ≥ 3 cm was an important indicator of poor prognosis in patients aged ≥50 years with PTC (7). Subsequently, the authors reported that in patients with high-risk PTC without distant metastasis, N ≥ 3 cm, together with age ≥55 years, tumor size >4 cm, and massive extrathyroidal extension, were significantly associated with DSS (although the extent of thyroidectomy was unrelated to patient prognosis) (24). In 2012, Ito *et al*. analyzed 5768 patients with PTC and reported that N ≥ 3 cm had the strongest prognostic impact on lymph node, lung, and bone recurrences (25). Furthermore, N ≥ 3 cm was independently related to DSS and OS in patients with PTC (17). Therefore, in those aged ≥55 and <55 years, the investigators proposed the upstaging of M0 PTC with N ≥ 3 cm from stage II to stage III and stage I to stage II, respectively (26). The risk classification system recommended by the Japan Association of Endocrine Surgeons (JAES) classified patients with N ≥ 3 cm and N < 3 cm into high-risk and intermediate-risk categories, respectively (27). Another important N characteristic, extranodal tumor extension, is described in the ‘Extranodal tumor extension (LNEx)’ subsection of the ‘Factors based on intraoperative findings’ section.

#### Distant metastasis at diagnosis (M1)

To date, numerous reports have been published on M1, which indubitably is an important prognostic factor for FTC and PTC. Despite the small sample size, one study showed that a primary lesion >4 cm, age ≥55 years, and massive extrathyroidal extension of the primary lesion independently affected the DSS of M1 patients (28). This indicates that the biologically aggressive characteristics of the primary lesions and the patient background significantly affect prognosis in M1 patients. Although, in this series, the RAI uptake was unrelated to the DSS, a more recent large-sample study by the same investigators showed that RAI-refractory disease was an independent predictor of cancer-related mortality in patients who had DTC with distant metastasis/recurrence (29). Furthermore, in patients with distant recurrences, age ≥55 years and massive extrathyroidal extension were independent predictors of cancer-related mortality (30). Furthermore, Lee *et al*. showed that extensive extrathyroidal extension of the primary lesion resulted in poor outcomes in PTC with initial distant metastasis (31). Furthermore, Matsuzu *et al*. reported higher mortality rates in patients with distant metastasis other than to the lungs, or lung metastasis with ≥2 risk factors: age ≥55 years, RAI-refractory distant metastasis, and surgical non-curative conditions(32). However, there are limitations to the evaluation of the prognosis of patients with distant metastasis/recurrence based only on static prognostic markers. Notably, evaluations that use dynamic prognostic factors are important, and even essential, for accurately predicting the prognosis of patients with distant metastasis or recurrence (as stated in the ‘Dynamic prognostic factors of PTC and FTC’ section). Furthermore, the recent and future development of molecular-targeted medicines may significantly contribute to changes in the prognostic impact of distant metastasis and recurrence step-by-step.

### Factors based on intraoperative findings

#### Extrathyroidal extension of PTC tumors

Extrathyroidal extension is an important predictive factor for the prognosis of PTC. In the present version of the TNM classification (1), extrathyroidal extension is reflected in T factor levels and is classified as follows: T3b, gross extrathyroidal extension invading only strap muscles (e.g. extension to sternothyroid, sternohyoid, thyrohyoid, or omohyoid muscles); T4a, gross extrathyroidal extension invading subcutaneous soft tissues, larynx, trachea, esophagus, or recurrent laryngeal nerve; and T4b, gross extrathyroidal extension invading prevertebral fascia or encasing the carotid artery or mediastinal vessels. Curative surgery of T4b is often difficult, and even though it is possible, Moritani *et al*. reported a dire prognosis for stage IVA disease (T4b and ≥55 years) (33). Previous studies have shown controversial results regarding the prognosis of patients with T3b (34, 35, 36, 37), and recently, a study that enrolled 7811 M0 PTC patients with a median postoperative follow-up period of 10.0 years showed that in patients aged ≥55 years, the prognosis of stage II/T3b was significantly poorer than that of stage I and did not differ from that of stage III/T4a. However, stage II/T3b and stage III/T4a1 had significantly better prognoses than stage III/T4a2 (See below on T4a1 and T4a2) (38).

T4a is the most common and important type of tumor invasion. In the TNM classification, patients with T4aM0 aged ≥55 years are classified with stage III disease. However, the prognosis of T4a carcinoma differs according to the depth of PTC invasion. Notably, Ito *et al*. classified T4a into two categories (T4a1 and T4a2) according to the organs to which the PTC invades, as shown in Table 2. They reported that the DSS rate of patients with stage III/T4a2 disease was significantly lower than that of patients with T4a1 disease (26). Further, the DSS of patients with stage III/T4a1 did not differ from that of patients with stage II disease who were ≥55 years of age. In the subset of patients aged <55 years, the DSS rate of patients with stage I/T4a2 was poorer than that of those without T4a2 and did not significantly differ from that of patients with stage II disease (M1 patients). The investigators proposed that patients aged ≥55 years with T4a1 were likely down-staged to stage II (the same stage as T3bM0), and those aged <55 years with T4a2 were likely upstaged to stage II. The general rules for the description of thyroid cancer by the Japan Association of Endocrine Surgery and the Japanese Society of Thyroid Pathology also divide T4a into two categories, similar, although not identical, to Ito’s classification (39).

**Table 2:** Ito’s subclassification of T4a. Based on Ito *et al.* (26).

Classification	Organs invaded by PTC
T4a1	Tracheal adventitia and/or cartilage Esophageal muscle layer Recurrent laryngeal nerve Cricothyroid and inferior pharyngeal constrictor muscles
T4a2	Subcutaneous soft tissues Larynx Tracheal mucosa Esophageal mucosa Jugular vein Brachiocephalic vein Sternocleidomastoid muscle

PTC, papillary thyroid carcinoma.

### Extranodal tumor extension (LNEx)

PTC invasion occurs not only in primary lesions but also in metastatic lymph nodes. Moritani reported that although the DSS rate of patients with LNEx-positive PTC was poorer than that of patients with LNEx-negative PTC, LNEx was not regarded as an independent prognostic factor for carcinoma death (40). In contrast, in 2007, Ito *et al*. showed that LNEx significantly affected DFS and DSS, in both univariate and multivariate analyses (41). In 2018, LNEx was also reported to independently impact the OS of patients (26). Thereafter, studies published in other countries have reported the prognostic significance of LNEx in PTC (42, 43, 44, 45, 46). For example, LNEx was reported to be associated with recurrence (41) and lung metastasis (46). Furthermore, Suh *et al*. reported that LNEx should be considered a poor prognostic marker in their meta-analysis (44), and Kim *et al*. claimed that the incorporation of LNEx in the ATA classification improves the accuracy of risk stratification for patients with thyroid carcinoma (45). Additionally, Ito *et al*. also reported that patients with LNEx-positivity are appropriate to be upstaged to stage II when <55 years and to stage III when ≥55 years old (47). In the risk classification of PTC conducted by the Japan Association of Endocrine Surgery, LNEx was adopted as a high-risk feature (27). Recently, a few studies have reported the prognostic value of LNEx (48, 49, 50). des (39).

### Factors based on postoperative findings

#### Aggressive variants

The pathological diagnosis of PTC and its subtypes is important for predicting the prognosis of PTC. To date, some aggressive variants have been identified, including tall cell, columnar cell, and hobnail variants (52, 53, 54). In the latest WHO classification, a new disease classification has been established, namely, high-grade follicular cell-derived non-anaplastic thyroid carcinoma, which is subdivided into poorly differentiated thyroid carcinoma and differentiated high-grade thyroid carcinoma (51). This type of malignancy was reported to be rare, at 1–6.7% of all thyroid carcinomas, with its incidence varying according to countries but showing a very dire prognosis (55). Clinicians should treat patients diagnosed with such variants carefully, even if no other high-risk features can be detected.

### Cell proliferating activity (Ki-67 labeling index (LI))

In 2010, Ito *et al*. showed that high Ki-67 LI is associated with both DFS and DSS in patients with PTC (56). Thereafter, several studies have reported the prognostic value of Ki-67 LI alone or in combination with other factors (57, 58, 59, 60, 61, 62). For example, Matsuse *et al*. showed that the combination of high Ki-67 LI (cutoffs set at 5 and 10%) and *TERT* promoter mutations (stated later) keenly reflect patient DFS (57). Additionally, Miyauchi *et al*. reported that a high Ki-67 LI was significantly associated with short thyroglobulin-doubling time (stated later). The authors concluded that the evaluation of Ki-67 LI in primary tumors may allow the prediction of postoperative thyroglobulin status, thyroglobulin-doubling time, and prognosis of PTC (58).

### *TERT* promoter mutations

To date, a few genetic mutations such as *BRAF*,* RET* fusion, and *NTRK* fusion mutations have been detected, and molecular-targeted medicines for these genes are available for advanced thyroid carcinoma therapy. However, the most important gene mutations that affect the prognosis are* TERT* promoter mutations. In 2014, Xing *et al*. reported that both* BRAF* V600E and *TERT* promoter mutations were predictors of PTC recurrence (63). Since then, a few other studies have investigated *BRAF* mutations and *TERT* promoter mutations; however, *BRAF* mutations were observed at high incidence and none of these studies showed that *BRAF* mutations alone significantly affect patients’ prognoses. In 2020, Ebina *et al*. showed that patients with PTC with *TERT* promoter mutations had poorer CSS than those without mutations (10-year CSS rates 73.7% vs 98.1%, and 10-year DFS rates 53.7% vs 93.3%). Notably, *TERT* promoter mutations are independent predictors of carcinoma mortality and recurrence. The investigators also showed that the DFS and DSS of patients with intrathyroidal PTCs that measured 1.1–4 cm without *TERT* promoter mutations, who underwent lobectomy, did not differ from those of patients who underwent total thyroidectomy (64). The researchers speculated that total thyroidectomy for PTCs measuring 1.1–4 cm may result in overtreatment if *TERT* promoter mutations are negative. Additional studies have been published on *TERT* promoter mutations and prognosis (54, 65, 66). Notably, Nakao *et al*. showed that preoperative detection of *TERT* promoter mutations using fine-needle aspiration is useful for predicting disease aggressiveness and determining PTC management strategy (67).

### Brief summary

PTC can be diagnosed based on preoperative cytological examination. Since it often metastasizes to regional lymph nodes and invades adjacent organs, pre- and intra-operative evaluations are very important for deciding the surgical design (extent of thyroidectomy and prophylactic/therapeutic lymph node dissection) and whether postoperative adjuvant therapy, such as RAI administration, should be performed. In addition, pathological examination to accurately diagnose aggressive variants is important for predicting the recurrence of carcinoma and deciding how clinicians should follow patients postoperatively. Furthermore, if available, Ki-67 LI evaluation and molecular testing, including *BRAF* gene mutation and *TERT* promoter gene mutation analysis, could be considered, which may be helpful in predicting patient prognosis. Table 3 summarizes the static prognostic factors of PTC based on pre-, intra-, and post-operative findings. There are differences in prognostic factors according to the periods when they are evaluated, and clinicians should accurately evaluate the outcomes of patients in each period.

**Table 3:** Static prognostic factors of PTC based on preoperative and intraoperative findings

**Findings/Factors**	**Endpoint of prognosis**
Preoperative findings	
Young age (see Table 1)	DFS
Old age (see Table 1)	DFS, DSS, OS
Male sex	DFS, OS
Tumor size >4 cm	DFS, DSS, OS
N < 3 cm	DFS
N ≥ 3 cm	DFS, DSS
M1	CSS
Intraoperative findings	
T4a1 (see Table 2)	DFS (≥55 years)
T4a2 (see Table 2)	DFS, DSS
T4b	DFS, DSS
LNEx	DFS, DSS, OS
Postoperative findings	
Aggressive variants	DFS, DSS
High Ki-67 LI	DFS, DSS

DFS, disease-free survival; DSS, disease-specific survival; LI, labeling index; LNEx, extranodal tumor extension; OS, overall survival; PTC, papillary thyroid carcinoma.

## Static prognostic factor of FTC

### Factors based on preoperative findings

Similar to PTC, some prognostic factors based on preoperative findings, such as patient age, tumor size, and M factor, have been reported. A meta-analysis of 2075 patients from 13 studies published in 2023 found that age >45 years, male sex, tumor diameter >4 cm, multifocality, and distant metastasis at diagnosis were prognostic factors for carcinoma death (68). The most recent single-institution study also showed that nodule size >40 mm and M factor were independent prognostic factors for DSS (69). Regarding age, Ito *et al*. divided patient age into three categories; <20 years, 20–44 years, and ≥45 years. In the subsets of both of minimally invasive FTC and widely invasive FTC (based on the latest edition of WHO classification), DFS and DSS were the poorest in patients ≥45 years. Although this study enrolled only 12 patients <20 years, their DFS was poorer than those aged 20–44 (70) This trend was similar to that observed for the PTC. More recently, Yamazaki *et al*. showed that in patients with minimally invasive FTC, a cutoff age of 55 years better reflected patient prognoses than that of 45 years (71), and Ito *et al*. showed that age ≥55 years was an independent predictor of FTC distant recurrence (72).

### Factors based on postoperative findings

#### Pathological findings: capsular and vascular invasions

The most recent WHO classification divides FTC into three categories based on capsular invasion (CI) and vascular invasion (VI): minimally invasive FTC (minimal CI only (detected only microscopically)), encapsulated angioinvasive (VI present with or without capsular penetration), and widely invasive FTC (extensive CI detected grossly) (55). Notably, the presence of VI is not required for the diagnosis of widely invasive FTC.

As for the prognostic impact of VI, Ito *et al*. showed that the number of VI events significantly affected distant recurrence (the number of VI, 0 *vs.* 1-3, vs ≥4) (72). Furthermore, Yamazaki *et al*. reported that the DFS, distant recurrence-free survival (DMFS), and DSS became poorer from VI = 0, VI = 1, to VI ≥ 2, but the prognoses did not differ between patients with VI = 1–3 and those with VI ≥ 4 (71). In a multivariate analysis, VI ≥ 2 was found to be an independent prognostic factor for DFS and DMFS. Further studies are necessary to set the optimal cutoff for the VI number; however, the prognosis of encapsulated angioinvasive FTC is not uniform but differs according to the degree of VI.

Ito *et al*. showed that the degree of CI (wide and minimal CI) did not affect DMFS of patients with FTC, because the DMFS of minimally invasive FTC and that of VI-negative widely invasive FTC did not significantly differ in their series (72). Furthermore, Yamazaki *et al*. reported an excellent prognosis of FTC with VI foci <2 (73) and subsequently showed that the DFS and DMFS of patients with VI-negative widely invasive FTC were similar to those of patients with minimally invasive FTC (74). Generally, widely invasive FTC is diagnosed based on the gross CI. Patients with FTC with a more extensive CI, for example, with significant invasion to adjacent organs, may have a poorer prognosis, but such incidences are very rare and, if they do occur, may be diagnosed as poorly differentiated carcinoma rather than FTC. Further investigation should be conducted to address this issue, but at least at present, the risk stratification of FTC is better based on VI than on CI, and the definition of widely invasive FTC should be reconsidered.

### Ki-67 LI

Similar to PTC, the prognostic impact of Ki-67 LI on FTC has been previously reported. Ito *et al*. showed that high Ki-67 LI (≥5%) significantly affected DFS in patients with minimally invasive FTC and widely invasive FTC, using the former WHO classification criteria (75, 76). Furthermore, Hellgren *et al*. reported that the Ki-67 LI in FTC was significantly higher than that in follicular adenoma (FA); however, the LI did not significantly differ among FTC subgroups. The investigators showed that a high Ki-67 LI was an independent predictor of both carcinoma recurrence and death, together with patient age (77). Thus, an optimal Ki-67 LI cut-off of 4% was suggested, based on the ROC curve.

#### Brief summary

In contrast to PTC, most FTCs are diagnosed via postoperative pathological examination. Because most patients undergo hemithyroidectomy, a second surgery (completion total thyroidectomy) should be performed for patients with a poor prognosis; therefore, pathological examination is important. A second surgery is unnecessary and should not be performed for minimally invasive FTC, and patients with encapsulated angioinvasive FTC with extensive VI (at least ≥4 invasive foci) should undergo complete total thyroidectomy to allow RAI administration with the goal of decreasing the risk of distant recurrence. The prognosis of widely invasive FTC is twofold: poor in VI-positive patients and excellent in VI-negative patients. Total thyroidectomy and successive RAI administration are necessary for VI-positive patients; however, based on two Japanese studies, hemithyroidectomy should be adequate for VI-negative patients. Further studies and reports from other countries are needed to address this issue. Table 4 summarizes the static prognostic factors for FTC.

**Table 4:** Static prognostic factors of FTC based on preoperative and postoperative findings

**Findings/ Factors**	**Endpoint of prognosis**
Preoperative findings	
Young age (<20 years)	DFS
Old age (≥45 or 55 years)	DFS, DSS
Male sex	DFS
Tumor size >4 cm	DFS, DSS
M1	DSS
Postoperative findings	
Vascular invasion (≥2 or ≥4)*	DFS, DSS
High Ki-67 LI	DFS

*Capsular invasion does not affect the prognosis of FTC. DFS, disease-free survival; DSS, disease-specific survival; FTC, follicular thyroid carcinoma; LI, labeling index.

## Dynamic prognostic factors of PTC and FTC

In contrast to the static factors, a few dynamic factors have prognostic significance. The values of these factors change over time, and an evaluation of their changing values is useful for predicting patient outcomes.

### Thyroglobulin-doubling rate (Tg-DR)/ thyroglobulin-doubling time (Tg-DT)

In 1984, Miyauchi *et al*. reported that the rate of increase in plasma calcitonin concentration, evaluated as calcitonin-doubling time (DT), was associated with survival in patients with medullary thyroid carcinoma (78). In 2011, the investigators found that short Tg-DT was significantly related to DSS in patients who underwent total thyroidectomy for PTC if their TgAbs were negative (79). Although the calculation formula is complicated, the Tg-DT calculator can be downloaded from the HP of Kuma Hospital (https://www.kuma-h.or.jp/kumapedia/kuma-medical/detail/?id=290). Notably, DT is a well-validated method for analyzing and expressing changes in tumor volume or serum tumor marker levels over time; however, it has two major limitations: if some patients show a decrease in tumor volume over time, their DTs are given negative values. This creates a discontinuity problem with the DTs of positive values. Secondly, the DT values are opposite to growth rate values, and an inverse DT value (1/DT) resolves this limitation. Miyauchi *et al*. named this index the doubling rate (DR) (80), which proved useful for evaluating changes in tumor volume over time in a cohort that included patients with decreased tumor volume over time. The DR indicates the number of doubling or halving events that occur per unit time.

### Tumor volume-doubling rate (TV-DR)/ tumor volume-doubling time (TV-DT)

Similar to Tg, the change in TV of metastatic lesions has a prognostic impact on DSS and should be calculated for metastatic lesions that develop rapidly. The maximum diameter (D1) and the diameter in the direction perpendicular to the maximum diameter (D2) are measured, and the tumor volume is calculated using the formula: (π/6 × D1 × D2 × D2). The formula for TV-DT has been described previously (80), and the calculation software can be downloaded as described above. In 2017, Sabra *et al*. showed that the TV-DT of distant metastatic lesions was significantly related to DSS in patients with DTC (81). In 2021, Ito *et al*. showed that high TV-DR (>1/year), together with Tg-DR >1/year, was an independent predictor of cancer-related death in patients with DTC with distant metastasis (29).

### Neutrophil-to-lymphocyte ratio

The neutrophil-to-lymphocyte ratio (NLR) is an inflammatory marker that reflects the imbalance between immune surveillance and tumor progression; an increased number of neutrophils and a decreased lymphocyte ratio are thought to reflect carcinoma progression and immunological surveillance, respectively (82, 83, 84). NLR has bilateral characteristics, as a static and dynamic prognostic factor. The prognostic impact of a high NLR in thyroid carcinomas has been investigated, but the study designs and results are rather fragmentary (82, 83, 84, 85, 86, 87, 88, 89, 90). In 2021, a study showed that NLR >3 at the time of detection of the distant recurrence of thyroid carcinoma was an independent predictor of CSS in patients with DTC (29). Furthermore, the investigators reported that the 5-year and 10-year DSS rates of patients with DTC with distant metastasis after NLR > 3 were low, at 50.4% and 23.9%, respectively. Additionally, Lee *et al*. showed that a significant increase in NLR was an independent risk factor for incomplete response to therapy (91); however, the NLR may change for reasons other than cancer status, and this limits its use.

Furthermore, a change in the NLR is useful for evaluating the effects of postoperative therapies for recurrent lesions. Ito *et al*. showed that changes in not only Tg levels but also NLRs of patients with DTC who underwent sorafenib or lenvatinib treatment strongly reflected the therapeutic efficacy (92). Additionally, Fukuda *et al*. showed that patients with DTC with a median NLR < 3 at the initiation of lenvatinib therapy had a longer OS and that the median NLR values decreased when the best tumor response was achieved, whereas they increased again at disease progression (93).

#### Brief summary and comments

Dynamic factors are very useful for estimating the recurrence and disease progression of patients during postoperative follow-up and for deciding if and when any treatment intervention is necessary. Both Tg-DR and TV-DR < 1 significantly predict poor patient DSS (28, 79). These parameters provide an indication for the treatment of recurrent lesions, such as external beam radiotherapy and molecular targeted therapies.

## Conclusions

In this review, we discussed the pre-, intra-, and post-operative prognostic factors of PTC and FTC. Pre- and intra-operative factors are useful for planning surgical design and postoperative management, including adjuvant treatment. Postoperative findings should be evaluated carefully to avoid oversight of important pathological or molecular findings. During postoperative follow-up, clinicians should consider changing dynamic factors to appropriately implement adjuvant/therapeutic RAI administration and, if needed, molecular-targeted therapy.

## Declaration of interest

The authors declare that there is no conflict of interest that could be perceived as prejudicing the impartiality of the study reported.

## Funding

This research did not receive any specific grant from any funding agency in the public, commercial, or not-for-profit sector.
